# Association of thyroid transcription factor‐1 with the efficacy of immune‐checkpoint inhibitors in patients with advanced lung adenocarcinoma

**DOI:** 10.1111/1759-7714.14560

**Published:** 2022-07-08

**Authors:** Kenji Nakahama, Hiroyasu Kaneda, Masahiko Osawa, Motohiro Izumi, Naoki Yoshimoto, Akira Sugimoto, Hiroaki Nagamine, Koichi Ogawa, Yoshiya Matsumoto, Kenji Sawa, Yoko Tani, Shigeki Mitsuoka, Tetsuya Watanabe, Kazuhisa Asai, Tomoya Kawaguchi

**Affiliations:** ^1^ Department of Respiratory Medicine, Graduate School of Medicine Osaka City University Osaka Japan; ^2^ Department of Clinical Oncology, Graduate School of Medicine Osaka Metropolitan University Osaka Japan; ^3^ Department of Diagnostic Pathology, Graduate School of Medicine Osaka Metropolitan University Osaka Japan; ^4^ Department of Pulmonary Medicine Bell land General Hospital Sakai Japan; ^5^ Department of Pulmonary Medicine Ishikiriseiki Hospital Higashiosaka Japan; ^6^ Department of Respiratory Medicine, Graduate School of Medicine Osaka Metropolitan University Osaka Japan

**Keywords:** adenocarcinoma, immunotherapy, non‐small cell lung cancer, programmed death‐ligand 1, thyroid transcription factor‐1

## Abstract

**Background:**

We aimed to identify the relationship between thyroid transcription factor‐1 (TTF‐1) expression of lung adenocarcinoma and the efficacy of immune‐checkpoint inhibitor (ICI) therapy.

**Methods:**

This retrospective multicenter study comprised patients with advanced lung adenocarcinoma treated with ICI monotherapy. We collected clinical medical records including data on TTF‐1 expression and analyzed the relationship between TTF‐1 expression and programmed death‐ligand 1 tumor proportion score (PD‐L1 TPS), objective response rate (ORR), progression‐free survival (PFS), and overall survival (OS).

**Results:**

In total, 108 patients with lung adenocarcinoma were analyzed. The rate of TPS ≥1% and ≥50% in patients with positive TTF‐1 expression was significantly higher than that in patients with negative TTF‐1 expression (88% vs. 60%, *p* < 0.001; 65% vs. 24%, *p* < 0.001). The ORR was significantly higher in TTF‐1 positive patients than in TTF‐1‐negative patients (38% vs. 8%, *p* = 0.003). Among patients with TPS ≥50% and 1%–49%, the ORR in TTF‐1 positive and negative patients was 48% (26/54) versus 17% (1/6) (*p* = 0.21), and 32% (6/19) versus 11% (1/9) (*p* = 0.37), respectively. The ORR for patients with TPS <1% was 0% in both the TTF‐1 negative and positive cases. The median PFS and OS was significantly longer in TTF‐1‐positive patients than in TTF‐1‐negative patients (5.4 vs. 1.6 months, *p* < 0.001; 18.2 vs. 8.0 months, *p* = 0.041). Multivariate analysis revealed that TTF‐1‐negative status was an independent unfavorable prognostic factor for PFS.

**Conclusion:**

Patients with TTF‐1‐positive status receiving ICI monotherapy showed better outcomes than those with TTF‐1‐negative lung adenocarcinoma.

## Graphical abstract

This multicenter retrospective study investigated the association between immune‐checkpoint inhibitor monotherapy effectiveness and the expression status of thyroid transcription factor 1 (TTF‐1) in 108 patients with advanced lung adenocarcinoma and showed that programmed death‐ligand 1 tumor proportion score, objective response rate, progression‐free survival, and overall survival were significantly lower in patients with TTF‐1‐negative expression than those in TTF‐1‐positive patients.

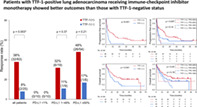

## INTRODUCTION

Thyroid transcription factor 1 (TTF‐1) is predominantly expressed in thyroid follicular cells and type II alveolar epithelial cells.^1^, ^2^ TTF‐1 regulates the maturation and development of thyroid and lung, and many studies have shown a relationship between TTF‐1 expression and the occurrence of lung cancer.^3^, ^4^, ^5^ TTF‐1 is expressed in 69%–80% of cases of nonsquamous non‐small cell lung cancer (NSCLC), and is commonly used to diagnose the histological type of lung cancer and distinguish primary lung adenocarcinoma from other metastatic adenocarcinomas in pathological cases.^6^, ^7^, ^8^, ^9^

TTF‐1 expression has prognostic relevance in NSCLC. In NSCLC, patients with positive TTF‐1 expression showed longer overall survival (OS) than those with negative TTF‐1 expression.^10^, ^11^ Furthermore, TTF‐1 has been reported to influence the sensitivity of NSCLC cells to cytotoxic chemotherapy.^12^ For example, pemetrexed‐based chemotherapy, a standard treatment for nonsquamous NSCLC,^13^, ^14^, ^15^ was inferior to pemetrexed‐free regimens in lung adenocarcinoma patients with negative TTF‐1 expression.^16^ Additionally, nonsquamous NSCLC patients with negative TTF‐1 expression treated with docetaxel, another standard cytotoxic agent for NSCLC, showed a lower disease control rate and shorter OS than their counterparts with positive TTF‐1 expression.^17^

Recently, immune checkpoint inhibitors (ICIs) have improved the prognosis of advanced NSCLC.^18^, ^19^, ^20^, ^21^, ^22^, ^23^, ^24^ In the first‐line treatment of NSCLC, pembrolizumab has shown clinical efficacy both when administered as monotherapy to advanced NSCLC patients with programmed death‐ligand 1 (PD‐L1) tumor proportion score (TPS) ≥50% and ≥1%, and when administered in combination with platinum‐doublet chemotherapy to all NSCLC patients regardless of PD‐L1 expression in tumor cells. Similarly, atezolizumab, administered as monotherapy to NSCLC patients with high PD‐L1 expression in tumor or immune cells, or in combination with platinum‐based chemotherapy to all NSCLC patients regardless of PD‐L1 expression status, was more effective than the platinum‐based chemotherapy. Thus, treatment strategies using cancer immunotherapy have become very complicated. Although PD‐L1 and other biomarkers, such as tumor mutation burden or tumor‐infiltrating T cells, have also been developed^25^, ^26^ to predict therapeutic responses to ICI, their predictive accuracy is insufficient for clinical use. Little is known about the association between the TTF‐1 expression and efficacy of ICI. Hence, we conducted a multicenter retrospective study to investigate the significance of TTF‐1 expression in NSCLC patients treated with ICI monotherapy.

## METHODS

### Study design and patients

We conducted a retrospective multicenter study between December 2015 and July 2020 on patients with advanced lung adenocarcinoma treated with ICI monotherapy (e.g., pembrolizumab, nivolumab, and atezolizumab) at Osaka Metropolitan University Hospital, Ishikiriseiki Hospital, and Bell Land General Hospital. The protocol was approved by the institutional review boards and Ethics Committees of all participating institutions (approval number: Osaka Metropolitan University Hospital 2020–177, Ishikiriseiki Hospital 20–26, and Bell Land General Hospital 2020–020). Written informed consent was obtained from most of the patients for participation in the study, and for those who could not visit the hospitals again, informed consent was obtained in the form of an opt‐out option on the website.

Patients who had previously received durvalumab as maintenance therapy after chemoradiation, anticytotoxic T‐lymphocyte‐associated antigen 4 therapy, or cytotoxic chemotherapy in combination with ICI were excluded; further, patients with unknown data for expression status of TTF‐1 and/or PD‐L1 were also excluded.

### Data collection

We reviewed the medical records of patients, including age, sex, smoking status, Eastern Cooperative Oncology Group Performance Status (ECOG PS) at the time of initiating ICI, histological type, tumor node metastasis stage, molecular profiling for epidermal growth factor receptor (EGFR) mutation status, fusion status of echinoderm microtubule‐associated protein‐like 4 and anaplastic lymphoma kinase, type and treatment line of ICI, TTF‐1 expression, tumor proportion score (TPS) of PD‐L1, response to ICI, progression‐free survival (PFS), and OS. PD‐L1 TPS was recorded at five different expression levels (<1%, 1%–24%, 25%–49%, 50%–74%, ≥75%); if the PD‐L1 TPS results were indicated as a range, the lower limit was used. Tumor responses were estimated using the Response Evaluation Criteria in Solid Tumors (RECIST) version 1.1.^27^ PFS was calculated from the date of the first ICI administration until disease progression or death from any cause, and OS was calculated from the date of the first ICI administration until death from any cause. The cutoff date was March 31, 2022. TTF‐1 expression was obtained from clinical pathology reports, and the TPS of PD‐L1 was measured from formalin‐fixed tumor samples using the commercially available PD‐L1 IHC 22C3 pharmDx assay (Dako North America, Inc.) at each institution.

### Statistical analysis

We analyzed the association between TTF‐1 expression positivity and TPS of PD‐L1 expression, objective response rate (ORR), PFS, and OS. Fisher's exact test was used to examine the association between two categorical variables. The Kaplan–Meier method was used to estimate survival curves, and the log‐rank test was used to compare the differences between the groups. The Cox proportional hazards model was used to assess the independent value of variables on PFS and OS and to calculate hazard ratios and perform multivariate analyses including the following variables, which have a possible influence on ICI effectiveness: TTF‐1 expression, TPS of PD‐L1, smoking status, ECOG PS, treatment line, *EGFR* mutation status. *p* < 0.05 was considered to be statistically significant. All statistical analyses were conducted using the JMP statistical software program (version 13.2.1, SAS Institute Inc.).

## RESULTS

### Patient characteristics

**FIGURE 1:**
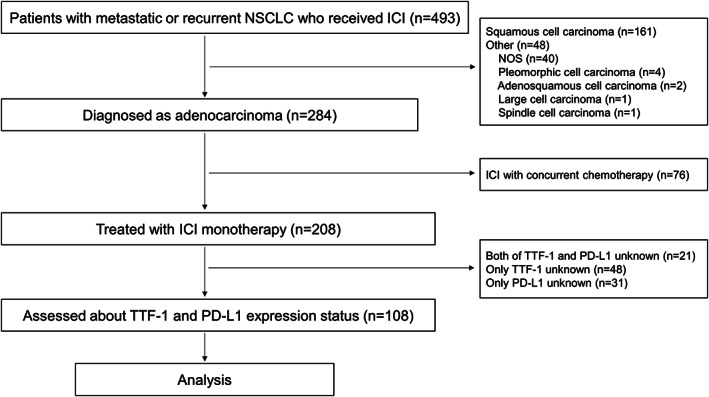
Study flow chart. NSCLC, non‐small cell lung cancer; ICI, immune‐checkpoint inhibitor; NOS, not otherwise specified; TTF‐1, thyroid transcription factor 1; PD‐L1, programmed death‐ligand 1

From the 493 advanced NSCLC patients who received ICI during the study period at the three institutions, 385 were excluded based on the following criteria: no adenocarcinoma (*n* = 209), received cytotoxic chemotherapy concurrent with ICI (*n* = 76), status of TTF‐1 and/or PD‐L1 missing (*n* = 100). Thus, a total of 108 patients were included in this study (Figure 1). TTF‐1 expression was present in 83 (77%) and absent in 25 (23%) patients. The median age at the time of ICI initiation was 72 (33–87) years, and 74 (69%) patients were male. Most patients (81%) had a history of smoking, and 85 (79%) had an ECOG PS of 0 or 1. There were 12 (11%) patients with *EGFR* mutation and one with anaplastic lymphoma kinase rearrangement. Seventy‐eight (72%) patients received pembrolizumab. The TTF‐1 positive rate was significantly higher in patients with PD‐L1 TPS ≥1% than in those with PD‐L1 TPS <1% (83% vs. 50%, *p* = 0.003) and was also significantly higher in patients with PD‐L1 TPS ≥50% than those with PD‐L1 TPS <50% (90% vs. 60%, *p* < 0.001) (Table 1).

**TABLE 1:** Patient characteristics

		TTF‐1 positive (*n* = 83)	TTF‐1 negative (*n* = 25)
Age	Median (range)	73 (33–87)	70 (52–84)
Sex	Male	60 (72)	14 (56)
	Female	23 (28)	11 (44)
Smoking status	Current or former smoker	70 (85)	16 (67)
	Never smoker	12 (15)	8 (33)
	Unknown	1 (1)	1 (4)
ECOG performance status score	0–1	65 (78)	20 (80)
	2 ≤	18 (22)	5 (20)
Stage	Stage III	17 (20)	0 (0)
	Stage IVA	30 (36)	12 (48)
	Stage IVB	29 (35)	10 (40)
	Postoperative recurrence	5 (6)	3 (12)
	Post‐radiotherapy recurrence	2 (2)	0 (0)
*EGFR* mutation status	Wild type	73 (88)	21 (84)
	Mutant	8 (10)	4 (16)
	Unknown	2 (2)	0 (0)
Type of ICI	Pembrolizumab	67 (81)	11 (44)
	Nivolumab	6 (7)	7 (28)
	Atezolizumab	10 (12)	7 (28)
ICI administration line	1	49 (59)	6 (24)
	2	22 (27)	10 (40)
	3 ≤	12 (14)	9 (36)
PD‐L1 tumor proportion score	<1%	10 (12)	10 (40)
	1%–49%	19 (23)	9 (36)
	≥50%	54 (65)	6 (24)

*Note*: Data presented as No (%).

Abbreviations: ECOG, Eastern Cooperative Oncology Group; EGFR, epidermal growth factor receptor; ICI, immune checkpoint inhibitor; PD‐L1, programmed death‐ligand 1; TTF‐1, thyroid transcription factor 1.

Analysis of positive TTF‐1 expression rate by PD‐L1 expression level showed an increasing trend for the TTF‐1 positive rate as PD‐L1 TPS increased (Figure S1).

### ORR of ICI treatment based on TTF‐1 and PD‐L1 expression status

**FIGURE 2:**
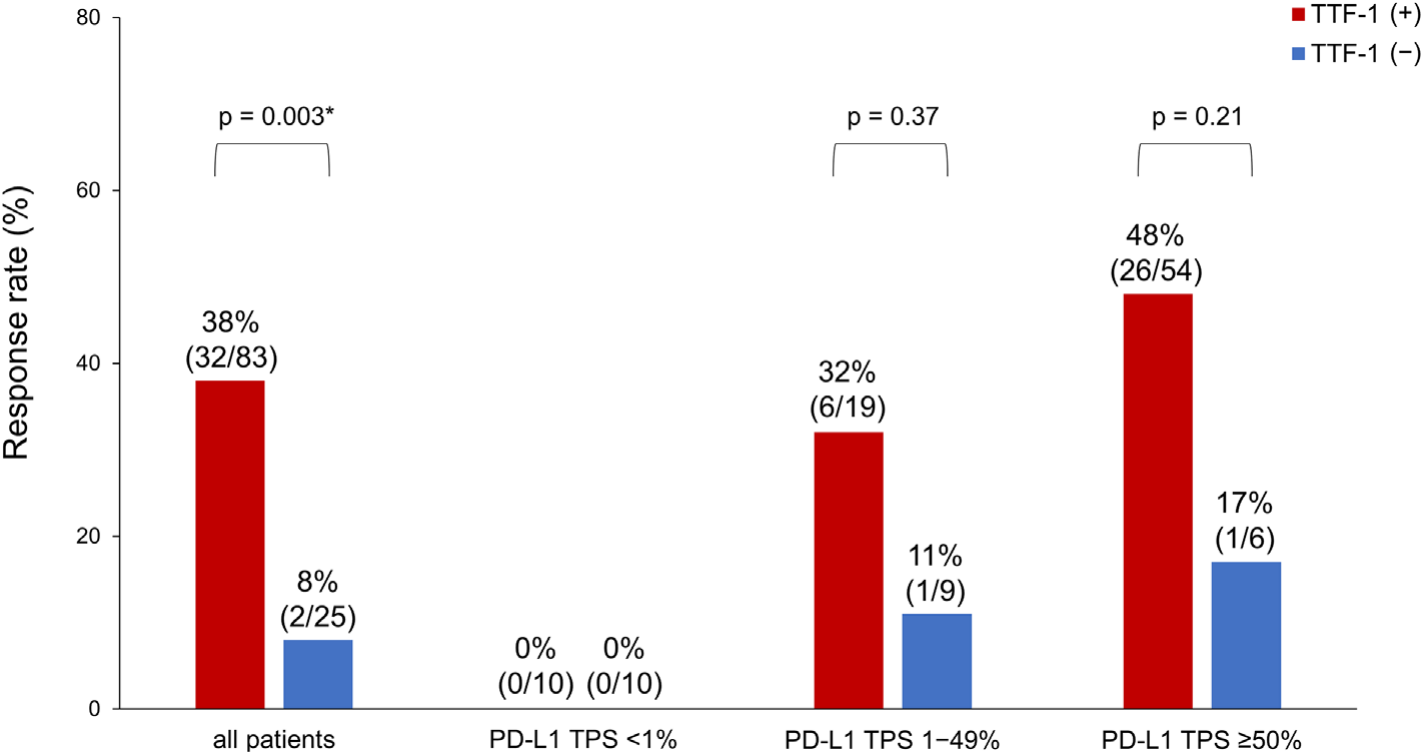
Objective response rate based on the status of thyroid transcription factor‐1 (TTF‐1) and programmed death‐ligand 1 (PD‐L1). TTF‐1, thyroid transcription factor 1; PD‐L1, programmed death‐ligand 1; TPS, tumor proportion score. *Indicates significant difference

The ORR was significantly higher in patients with positive TTF‐1 expression than in those with negative TTF‐1 expression (38% vs. 8%, respectively, *p* = 0.003) (Figure 2). When both TTF‐1 expression status and PD‐L1 TPS were considered in the analysis, among patients with PD‐L1 TPS ≥50%, the ORR was 17% (1/6) and 48% (26/54) in patients with negative and positive TTF‐1 status, respectively (*p* = 0.21). Among patients with PD‐L1 TPS 1%–49%, the ORR was 11% (1/9) and 32% (6/19) in patients with negative and positive TTF‐1 expression, respectively (*p* = 0.37). The ORR for patients with PD‐L1 TPS <1% was 0% in both the TTF‐1 negative and positive cases (Figure 2).

### PFS and OS based on status of TTF‐1 and PD‐L1 expression

**FIGURE 3:**
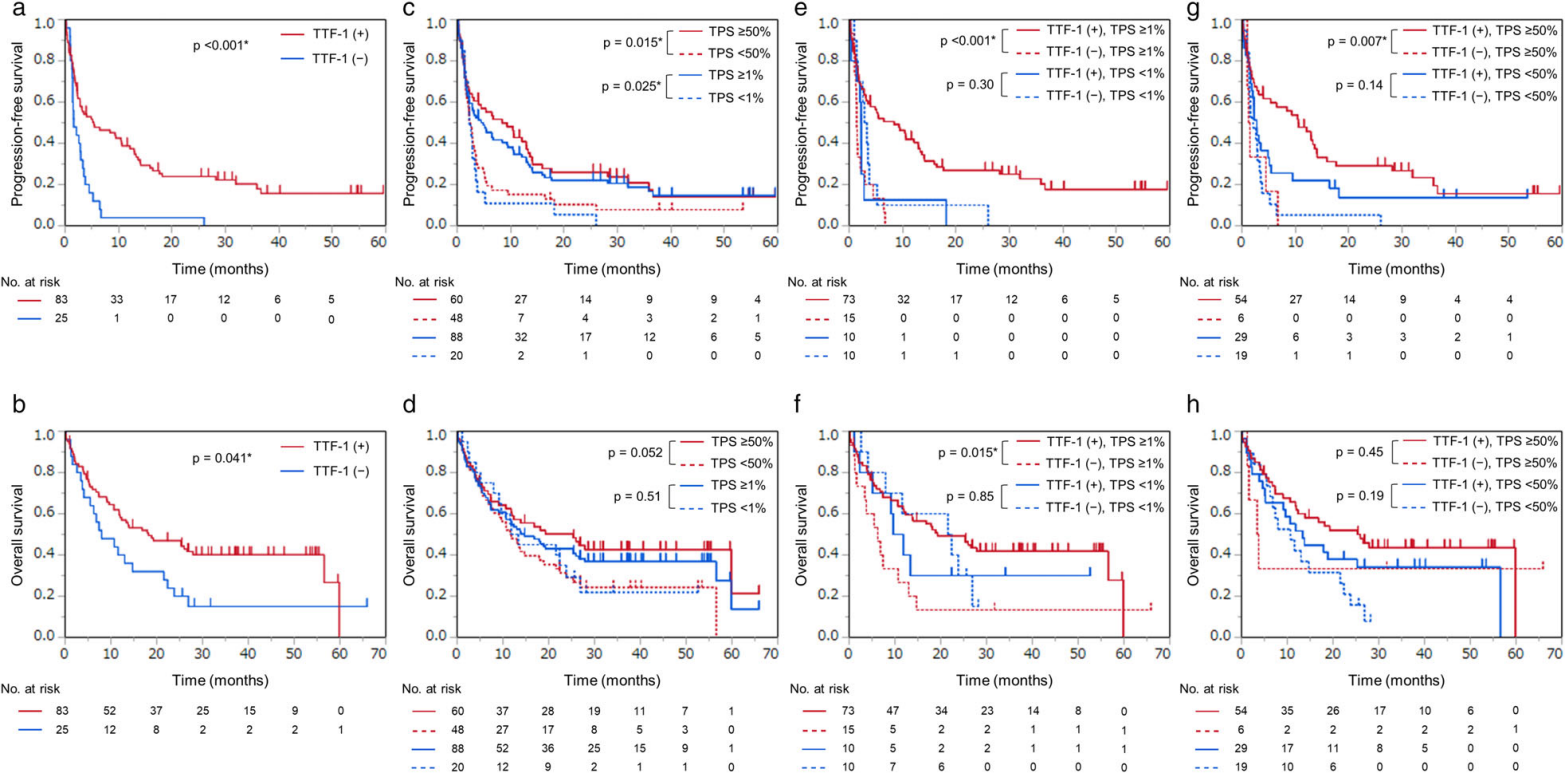
Progression‐free survival (PFS) and overall survival (OS) based on the status of thyroid transcription factor‐1 (TTF‐1) and programmed death‐ligand 1 (PD‐L1). PFS and OS were stratified according to TTF‐1 expression (A, B) and PD‐L1 expression (C, D) and in combination (E, F, G, H). TTF‐1, thyroid transcription factor‐1; TPS, tumor proportion score. *Indicates significant difference

The median PFS was significantly longer in patients with positive TTF‐1 expression than in those with negative TTF‐1 expression (5.4 vs. 1.6 months, *p* < 0.001) (Figure 3A). Likewise, the median OS was longer in patients with positive TTF‐1 expression than in their TTF‐1‐negative counterparts (18.2 vs. 8.0 months, *p* = 0.041) (Figure 3B). The median PFS was significantly longer in patients with PD‐L1 TPS ≥50% than in those with PD‐L1 TPS <50% (8.4 vs. 2.3 months, *p* = 0.015); moreover, the median PFS in patients with PD‐L1 TPS ≥1% and PD‐L1 TPS <1% was 4.9 and 2.3 months, respectively (*p* = 0.025) (Figure 3C). The median OS was 25.3 and 11.7 months in patients with PD‐L1 TPS ≥50% and PD‐L1 TPS <50%, respectively (*p* = 0.052), and it was 14.7 and 12.6 months in those with PD‐L1 TPS ≥1% and PD‐L1 TPS <1%, respectively (*p* = 0.51) (Figure 3D). Thereafter, we divided patients into four groups based on TTF‐1 and PD‐L1 status. Among patients with PD‐L1 TPS ≥1%, the median PFS was 8.4 and 1.4 months in those who were TTF‐1‐positive and TTF‐1‐negative, respectively (*p* < 0.001). Among patients with PD‐L1 TPS <1%, the median PFS was 2.2 and 3.1 months in those who were TTF‐1‐positive and TTF‐1‐negative, respectively (*p* = 0.30) (Figure 3E). Among patients with PD‐L1 TPS ≥1%, the median OS was 19.3 and 6.2 months in those who were TTF‐1‐positive and TTF‐1‐negative, respectively (*p* = 0.015). Among patients with PD‐L1 TPS <1%, the median OS was 10.7 and 22.0 months in those who were TTF‐1‐positive and TTF‐1‐negative, respectively (*p* = 0.85) (Figure 3F). Among patients with PD‐L1 TPS ≥50%, the median PFS was significantly longer in patients with positive TTF‐1 expression than those with negative expression (10.5 vs. 1.4 months, *p* = 0.007). The median PFS was 2.8 and 2.1 months in patients with positive and negative TTF‐1 expression with PD‐L1 TPS <50%, respectively. (*p* = 0.14) (Figure 3G). The median OS was 26.0 and 3.6 months in patients with positive and negative TTF‐1 expression with PD‐L1 TPS ≥50%, respectively (*p* = 0.45); further, it was 13.3 and 10.7 months in patients with positive and negative TTF‐1 expression with PD‐L1 TPS <50%, respectively (*p* = 0.19) (Figure 3H).

### PFS and OS analysis by ICI treatment line

Among patients who received ICI monotherapy as first‐line treatment, PFS and OS were significantly longer in patients with positive TTF‐1 expression than those with negative TTF‐1 expression (10.5 vs. 1.4 months, *p* = 0.048, 25.3 vs. 3.6 months, *p* = 0.035, respectively). (Figure S2A,B) Among patients treated with ICI as second or later line therapy, PFS was significantly longer in patients with positive TTF‐1 expression than in those with negative TTF‐1 expression (2.8 vs. 2.1 months, *p* = 0.032) whereas there was no significant difference in OS (12.8 vs. 11.5 months, *p* = 0.34) (Figure S2C,D).

### Univariate and multivariate analysis for PFS and OS

In the univariate analysis for PFS, the factors of TTF‐1 negative status, PD‐L1 TPS <50%, no history of smoking, ECOG PS ≥2, and second or later line of ICI treatment were significantly associated with poor PFS. Among these factors, TTF‐1 negative status and PS ≥2 were significantly associated with poor PFS in the multivariate analysis (*p* = 0.011, *p* < 0.001, respectively) (Table 2). For OS, no history of smoking and ECOG PS ≥2 were significantly associated with poor OS in the univariate analysis (*p* = 0.033, *p* < 0.001, respectively) and only ECOG PS ≥2 was significantly associated with poor OS in the multivariate analysis (*p* < 0.001) (Table 3).

**TABLE 2:** Univariate and multivariate analysis for progression‐free survival

			Median PFS (months)	Univariate analysis			Multivariate analysis		
Factor		N		HR	95% CI	*p*‐value	HR	95% CI	*p*‐value
TTF‐1	Positive	83	5.4						
	Negative	25	1.6	2.42	1.46–3.90	<0.001	2.09	1.19–3.57	0.011
PD‐L1 TPS	≥50%	60	8.4						
	<50%	48	2.3	1.68	1.10–2.57	0.017	1.13	0.64–2.03	0.67
Smoking status	Current or former smoker	86	4.5						
	Never smoker	20	1.6	1.95	1.12–3.21	0.020	1.59	0.85–2.86	0.14
ECOG PS	0–1	85	5.4						
	≥2	23	1.0	5.38	3.11–9.05	<0.001	6.49	3.60–11.42	<0.001
Treatment line	First‐line	55	9.3						
	Second or later	53	2.8	1.65	1.08–2.54	0.020	1.64	0.91–2.92	0.095
*EGFR* mutation	Wild‐type	94	3.3						
	Mutant	12	2.8	1.20	0.60–2.16	0.59	1.25	0.61–2.58	0.54

Abbreviations: CI, confidence interval; ECOG PS, Eastern Cooperative Oncology Group Performance Status; EGFR, epidermal growth factor receptor; HR, hazard ratio; PD‐L1, programmed death‐ligand 1; PFS, progression‐free survival; TPS, tumor proportion score; TTF‐1, thyroid transcription factor 1.

**TABLE 3:** Univariate and multivariate analysis for overall survival

			Median OS (months)	Univariate analysis			Multivariate analysis		
Factor		N		HR	95% CI	*p*‐value	HR	95% CI	*p*‐value
TTF‐1	Positive	83	18.2						
	Negative	25	8.0	1.70	0.99–2.80	0.052	1.57	0.87–2.76	0.13
PD‐L1 TPS	≥50%	60	25.3						
	<50%	48	11.7	1.59	0.99–2.56	0.054	1.80	0.92–3.59	0.088
Smoking status	Current or former smoker	86	18.9						
	Never smoker	20	5.7	1.92	1.06–3.29	0.033	1.88	0.97–3.44	0.061
ECOG PS	0–1	85	23.8						
	≥2	23	2.1	5.58	3.23–9.38	<0.001	6.63	3.69–11.62	<0.001
Treatment line	First‐line	55	19.3						
	Second or later	53	12.3	1.23	0.77–1.97	0.39	1.21	0.61–2.39	0.59
*EGFR* mutation	Wild‐type	94	16.8						
	Mutant	12	6.2	1.59	0.75–3.02	0.21	1.36	0.61–2.79	0.42

Abbreviations: CI, confidence interval; ECOG PS, Eastern Cooperative Oncology Group Performance Status; EGFR, epidermal growth factor receptor; HR, hazard ratio; OS, overall survival; PD‐L1, programmed death‐ligand 1; TPS, tumor proportion score; TTF‐1, thyroid transcription factor 1.

## DISCUSSION

In this study, TTF‐1‐positive patients with lung adenocarcinoma who received ICI monotherapy showed significantly higher ORR and longer PFS than their TTF‐1‐negative counterparts. Furthermore, to the best of our knowledge, this is the first report to analyze the relationship between TTF‐1 and PD‐L1 expression status based on PD‐L1 TPS ≥1% and ≥ 50% in advanced lung adenocarcinoma, and TTF‐1 expression status was significantly correlated with PD‐L1 expression status in advanced lung adenocarcinoma. Surprisingly, the positive rate of TTF‐1 was markedly high (97%) in the group expressing very high levels of PD‐L1 (PD‐L1 TPS≥75%).

The role of TTF‐1 may be associated with the occurrence of lung cancer.^5^, ^28^ TTF‐1 is expressed in 69%–80% of cases of lung adenocarcinoma,^6^, ^7^, ^8^, ^29^ and the amplification of TTF‐1 has been reported to lead to the proliferation of lung cancer cells.^30^, ^31^ Clinically, TTF‐1 expression is commonly used to diagnose the histological type of lung cancer and distinguish primary lung adenocarcinoma from other metastatic adenocarcinomas; additionally, it can be used as a prognostic marker.^29^ Patients with positive TTF‐1 expression showed longer OS than those with negative TTF‐1 expression in stage I lung adenocarcinoma.^11^ In advanced NSCLC treated with cytotoxic anticancer therapy, TTF‐1 expression was associated with prolonged survival and efficacy of anticancer therapy.^10^ Pemetrexed is regarded as one of the standard chemotherapies for advanced nonsquamous NSCLC.^13^, ^14^, ^15^ However, pemetrexed‐based regimen has been inferior to nonpemetrexed‐based chemotherapy in lung adenocarcinoma patients with negative TTF‐1 expression in terms of PFS and OS.^16^ Hence, the evaluation of TTF‐1 expression is important in determining the chemotherapy regimen for non‐squamous NSCLC. In clinical practice or trials, nonsquamous NSCLC is often classified as a diagnosis other than squamous cell carcinoma, and therefore, nonsquamous NSCLC includes clearly different histological types from adenocarcinoma, such as sarcomatoid carcinoma. In this study, we limited our analysis to adenocarcinoma to keep the histological background as similar as possible, which reduced the effect of confounding factors when evaluating the significance of TTF‐1 expression.

Recently, several reports have indicated that TTF‐1 expression is correlated with the efficacy of ICI and PD‐L1 expression. The analysis of 36 patients with sarcomatoid lung cancer revealed that the PD‐L1 expression rate was higher in patients with positive TTF‐1 expression than in those with negative TTF‐1 expression.^32^ The immunohistochemical analysis, using tissue microarrays of 866 NSCLC including 364 adenocarcinoma, showed a weak positive correlation between TTF‐1 and PD‐L1 expression.^33^ Among 231 patients with non‐squamous NSCLC who received ICI, better PFS and OS were observed in patients with positive TTF‐1 expression than in those with negative TTF‐1 expression; moreover, there was a weak positive association between TTF‐1 and PD‐L1 expression.^34^ However, there is a lack of data on TTF‐1 expression rate in NSCLC patients with PD‐L1 TPS ≥50% and the association between TTF‐1 expression and efficacy of ICI monotherapy in patients with lung adenocarcinoma only.

In this study, TTF‐1‐positive lung adenocarcinoma patients with both TPS ≥1% and TPS ≥50% of PD‐L1 expression had better clinical outcomes than those with negative TTF‐1 expression. It has been reported that TTF‐1 negative lung adenocarcinoma had more frequent Kelch‐like epichlorohydrin‐associated protein 1 (KEAP1) mutations, which negatively affect ICI efficacy, than TTF‐1 positive lung adenocarcinoma.^35^, ^36^ In preclinical models, TTF‐1 negativity is associated with loss of serine–threonine kinase 11 (STK11), which also negatively correlates with ICI effectiveness.^37^, ^38^, ^39^, ^40^ Furthermore, uncommon histological type of adenocarcinoma such as invasive mucinous adenocarcinomas and enteric adenocarcinomas frequently exhibited TTF‐1 negative status and those cancers are known to be less responsive to ICI.^41^, ^42^, ^43^ Although the present study did not assess the KEAP1 and/or STK11 gene alterations and the detailed types of adenocarcinomas, above‐mentioned reports might partially explain why TTF‐1 negative lung adenocarcinoma showed worse outcomes with ICI monotherapy in our study. Few reports have suggested the mechanism of correlation between TTF‐1 and PD‐L1. TTF‐1 induced receptor tyrosine kinase‐like orphan receptor 1, which regulates the phosphoinositide 3‐kinase/protein kinase B (PI3K/AKT) pathway, and activation of PI3K‐AKT can promote PD‐L1 expression.^44^, ^45^ On the contrary, TTF‐1‐negative lung adenocarcinoma cell lines overexpressed serglycin, which was reported to upregulate the PD‐L1 expression.^46^ Thus, the mechanism of the association between TTF‐1 and PD‐L1 is still unclear and requires further investigation.

Our results indicate that TTF‐1 expression had a meaningful association with both PD‐L1 expression and efficacy of ICI monotherapy in advanced lung adenocarcinoma, suggesting that, in addition to PD‐L1, the assessment of TTF‐1 as a biomarker before ICI treatment may help physicians choose appropriate regimens, such as ICI with or without cytotoxic agents. Given the low response rate of ICI monotherapy in patients without TTF‐1 expression in our study, the possibility of poor efficacy should be considered when treating TTF‐1 negative lung adenocarcinoma with ICI monotherapy. Future exploration of treatment options to improve outcomes in TTF‐1 negative patients, such as combination therapy with cytotoxic anticancer agents or other immunotherapies, is warranted.

This study has some limitations, including the retrospective nature of the study design. This study excluded adenocarcinoma patients whose TTF‐1 expression status was unknown; this could be a selection bias as TTF‐1 would be measured when morphological features of adenocarcinoma were not apparent. However, since the positive rate of TTF‐1 expression in this study is comparable to that previously reported,^6^, ^7^, ^8^, ^9^ we believe that the bias is not significant. Other limitation is the relatively small sample size in this study, particularly when divided into four groups based on TTF‐1 and PD‐L1 expression status.

In conclusion, we found that the positivity of TTF‐1 expression was associated with PD‐L1 expression status, and that adenocarcinoma patients with positive TTF‐1 expression showed better ORR and PFS than those with negative TTF‐1 expression when treated with ICI monotherapy. Regardless of PD‐L1 expression, caution is required when treating TTF‐1 negative patients with ICI monotherapy. Larger prospective trials investigating whether TTF‐1 expression influences the therapeutic effects of immunotherapy are warranted.

## CONFLICT OF INTEREST

H. Kaneda reports personal fees from MSD, Ono Pharmaceutical Co., Ltd.; Bristol‐Myers Squibb K.K. Chugai Pharmaceutical Co., Ltd.; outside the submitted work. S. Mitsuoka reports personal fees from MSD K.K., during the conduct of the study, and personal fees from Ono Pharmaceutical Co., Ltd.; Bristol‐Myers Squibb K.K.; Taiho Pharmaceutical Co., Ltd.; Kyowa Hakko Kirin Co., Ltd.; Lidye Co., Ltd.; Chugai Pharmaceutical Co., Ltd.; and AstraZeneca K.K., outside the submitted work. T. Kawaguchi reports personal fees from MSD, Novartis, and Pfizer; grants and personal fees from Ono Pharmaceutical Company, Chugai Pharmaceutical Company, AstraZeneca, Taiho Pharmaceutical Company, Boehringer Ingelheim Pharmaceutical, Bristol Myers Squibb, and Lilly; and grants from Kyowa Kirin, outside the submitted work. K. Sawa reports personal fees from Chugai Pharmaceutical Co., Ltd, personal fees from Nippon Boehringer Ingelheim Co., Ltd., personal fees from Daiichi Sankyo Company, Ltd, outside the submitted work. K. Nakahama, M. Osawa, M. Izumi, N. Yoshimoto, H. Nagamine, K. Ogawa, Y. Matsumoto, Y. Tani, T. Watanabe, and K. Asai have nothing to disclose.

## Supporting information


**Figure S1.** The positive thyroid transcription factor‐1 (TTF‐1) expression rate by programmed death‐ligand 1 (PD‐L1) expression level. TTF‐1, thyroid transcription factor 1; PD‐L1, programmed death‐ligand 1; TPS, tumor proportion score.

**Figure S2.** Progression‐free survival (PFS) and overall survival (OS) analysis by immune‐checkpoint inhibitor treatment line. TTF‐1, thyroid transcription factor 1.
