# Supplementary figures and images for: Association of thyroid transcription factor‐1 with the efficacy of immune‐checkpoint inhibitors in patients with advanced lung adenocarcinoma
Source: Thorac Cancer. 2022 Jul 8;13(16):2309–17. doi: 10.1111/1759-7714.14560 (PMC9376174; doi:10.1111/1759-7714.14560)

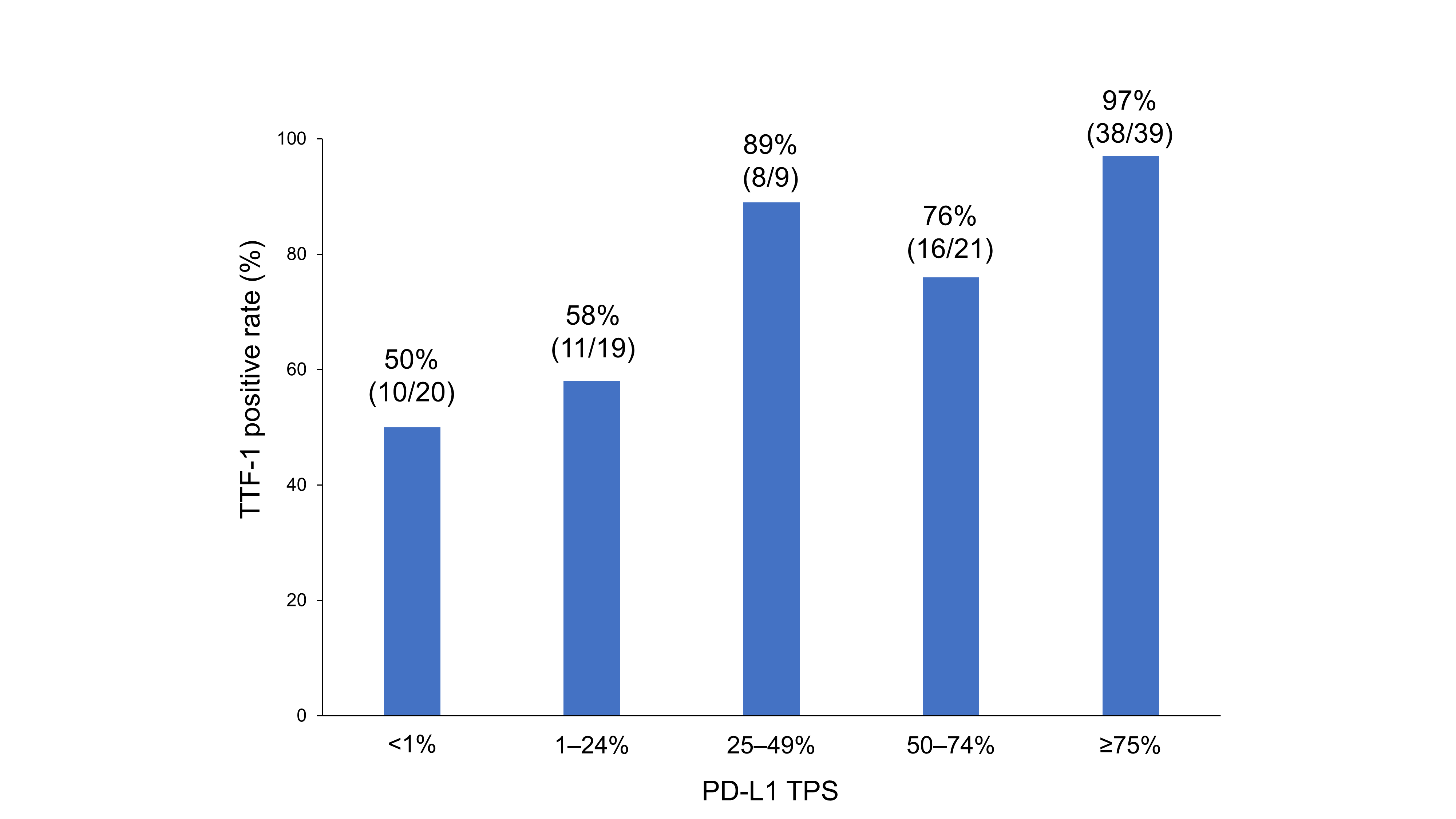

Supplement: Supplementary file 1 — Figure S1. The positive thyroid transcription factor‐1 (TTF‐1) expression rate by programmed death‐ligand 1 (PD‐L1) expression level. TTF‐1, thyroid transcription factor 1; PD‐L1, programmed death‐ligand 1; TPS, tumor proportion score. [file TCA-13-2309-s001.tif]

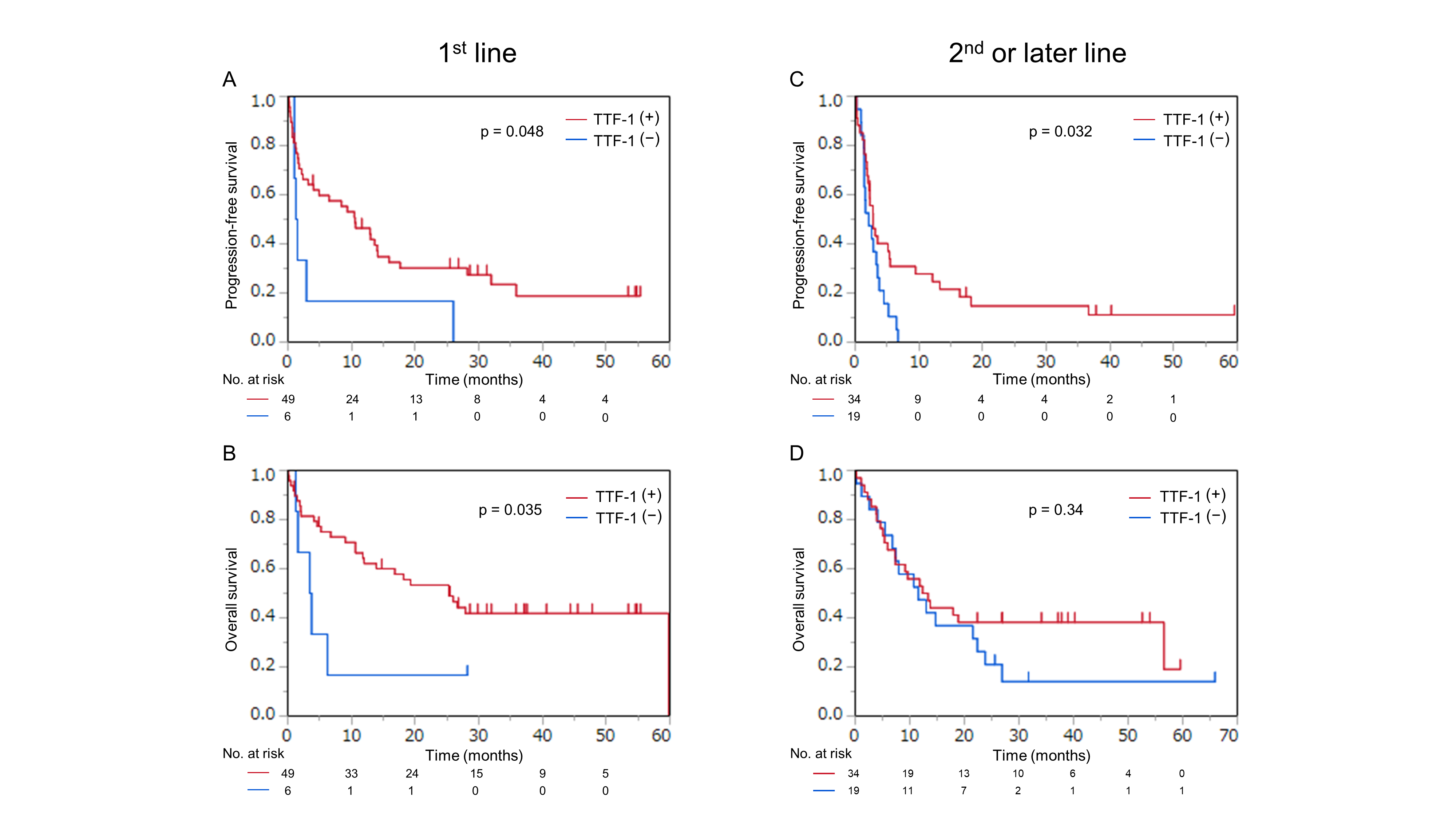

Supplement: Supplementary file 2 — Figure S2. Progression‐free survival (PFS) and overall survival (OS) analysis by immune‐checkpoint inhibitor treatment line. TTF‐1, thyroid transcription factor 1. [file TCA-13-2309-s002.tif]
